# Fabrication and Sintering Behavior of Er:SrF_2_ Transparent Ceramics using Chemically Derived Powder

**DOI:** 10.3390/ma11040475

**Published:** 2018-03-22

**Authors:** Jun Liu, Peng Liu, Jun Wang, Xiaodong Xu, Dongzhen Li, Jian Zhang, Xinming Nie

**Affiliations:** 1 Jiangsu Key Laboratories of Advanced Laser Materials and Devices, School of Physics and Electronics Engineering, Jiangsu Normal University, Xuzhou 221116, China; ljun2kmi@gmail.com (J.L.); jwang025@e.ntu.edu.sg (J.W.); xdxu79@mail.sic.ac.cn (X.X.); dzhl@siom.ac.cn (D.L.); nxinming@jsnu.edu.cn (X.N.); 2 Key Laboratory of Transparent Opto-Functional Inorganic Materials, Shanghai Institute of Ceramics, Chinese Academy of Sciences, Shanghai 200050, China; jianzhang@mail.sic.ac.cn

**Keywords:** co-precipitation method, Er:SrF_2_, transparent ceramics, densification, lattice diffusion

## Abstract

In this paper, we report the fabrication of high-quality 5 at. % Er^3+^ ions doped SrF_2_ transparent ceramics, the potential candidate materials for a mid-infrared laser-gain medium by hot-pressing at 700 °C for 40 h using a chemically-derived powder. The phase structure, densification, and microstructure evolution of the Er:SrF_2_ ceramics were systematically investigated. In addition, the grain growth kinetic mechanism of Er:SrF_2_ was clarified. The results showed lattice diffusion to be the grain growth mechanism in the Er:SrF_2_ transparent ceramic of which highest in-line transmittance reached 92% at 2000 nm, i.e., very close to the theoretical transmittance value of SrF_2_ single crystal. Furthermore, the emission spectra showed that the strongest emission band was located at 2735 nm. This means that it is possible to achieve a laser output of approximately 2.7 μm in the 5 at. % Er^3+^ ions doped SrF_2_ transparent ceramics.

## 1. Introduction

Mid-infrared lasers, operating roughly within the 2.5–3 μm spectral range, are a research focus in the solid-state laser field because of their potential applications in medicine, biological processing, remote sensing, and pollution monitoring [1,2,3,4]. The thermal effect [5], especially the thermal lens effect of laser gain medium materials [6], seriously deteriorates the output power and beam quality of mid-infrared solid-state lasers in operation. However, gain-medium materials with a negative thermo-optic coefficient can effectively compensate for the thermal lens effect [7], which can contribute to high output power in mid-infrared lasers. Since Ikesue et al. reported the Nd:YAG transparent ceramic as a solid-state laser gain medium. Since Ikesue reported the Nd:YAG transparent ceramic as a solid laser gain medium [8], transparent ceramics doped with rare earth ions [9], such as YAG [10,11,12], sesquioxide [13,14,15,16,17] and fluoride [18,19,20,21,22,23], have been extensively investigated. Compared with single crystal, transparent ceramics can be fabricated on a large scale, can be mass produced, and can be homogenously and heavily doped with active ions [24,25,26]. Furthermore, the mechanical strength of laser ceramics has also been enhanced [27]. Among laser ceramics, fluoride ceramics have a broad range of transmittance, low refraction indexes and lower phonon energy [28]. In particular, fluoride materials, such as CaF_2_ and SrF_2_, have a negative thermo-optical coefficient [29,30]. All of the aforementioned properties are suitable for mid-infrared laser output. 

In 1964, the first laser ceramic, Dy:CaF_2_, was fabricated by Hatch et al. [31]. However, progress in the development of fluoride laser ceramics was slow over the next few decades. At the beginning of the 20th century, Basiev et al. [27,32,33] used the hot forming method to successfully fabricate high quality transparent fluoride ceramics. Fluoride single crystals were used as the starting material, which were then deformed under the high temperature and high pressure in the mold. These deformed fluoride ceramics possessed similar laser properties compared to those of the fluoride single crystal. Moreover, the mechanical strength was evidently enhanced in the fluoride ceramics. This stemmed from the removal of the cleavage plane in the fluoride single crystal during the ceramization process under the high pressure and elevated temperature. Furthermore, Mortier et al. [34,35] developed a novel method of fabricating transparent high quality fluoride ceramics by sintering under a vacuum combined with a hot-isostatic pressing post-treatment using a co-precipitation powder. Mei et al. [36,37] fabricated fluoride transparent ceramics by vacuum hot-pressing or spark plasma sintering directly. Interestingly, Chen et al. [38] prepared transparent CaF_2_ nanocomposites by casting low-viscosity monomers with a ceramic green body. However, the microstructure evolution and densification of fluoride ceramics during sintering were rarely investigated in these studies, let alone the grain growth kinetic mechanism in fluoride ceramics. These factors can all contribute to the optical quality of fluoride ceramics, which can favor high laser outputs.

In this paper, the SrF_2_ ceramics doped with 5 at. % Er^3+^ ions were fabricated by hot-pressing under a vacuum at 10^−3^ Pa using chemically derived powder. SrF_2_ has a lower phonon energy and a smaller thermo-optical coefficient compared with CaF_2_. Moreover, the photon transitions of ^4^I_11/2_ → ^4^I_13/2_ in Er^3+^ ions can produce emission at approximately 2.6–3 μm. Thus, these properties in a SrF_2_ system doped with Er^3+^ ions can help achieve high laser output in the mid-infrared region. In order to optimize the optical quality of fluoride ceramics, the densification and microstructure evolution of the Er:SrF_2_ ceramics sintered at different temperatures were investigated in detail. In addition, the grain growth kinetic mechanism was clarified. Moreover, the in-line transmittance and photoluminescence emission spectra of the 5 at. % Er^3+^ doped SrF_2_ transparent ceramics were also studied in this paper.

## 2. Materials and Methods

### 2.1. Preparation of the Nanoparticles, Powder and Ceramics

Commercially available chemical reagents including strontium nitrate (Sr(NO_3_)_2_, AR, ≥99.5%, Sinopharm Chemical Reagent Co., Ltd., Shanghai, China), potassium fluoride dihydrate (KF·2H_2_O, AR, ≥99.0%, Sinopharm Chemical Reagent Co., Ltd., Shanghai, China), erbium oxide (Er_2_O_3_, 99.99%, Sinopharm Chemical Reagent Co., Ltd., Shanghai, China) and nitric acid (HNO_3_, GR, roughly 65.0–68.0%, Sinopharm Chemical Reagent Co., Ltd., Shanghai, China) were used as starting materials. For synthesis, a 1 M Er(NO_3_)_3_ stock solution was obtained by dissolving Er_2_O_3_ in HNO_3_ at an elevated temperature. The 1 M Sr(NO_3_)_2_ stock solution was prepared by dissolving the corresponding salt in deionized water. KF was dissolved in deionized water to prepare the 2 M stock solutions. The resistivity of the deionized water in the experiment was 18.25 MΩ·cm. Concentrations of every solution were quantified with a volumetric flask. For the synthesis of 5 at. % Er:SrF_2_, 20 mL of Er(NO_3_)_3_ (1 M) solution was mixed with 380 mL of Sr(NO_3_)_2_ (1 M) solution. Next, 420 mL of the KF (2 M) solution was added into the nitrate mixed solution using a peristaltic pump under magnetic stirring. The mixed suspension solution was maintained at room temperature for 24 h. The obtained nanoparticles were collected by centrifugation, washed with deionized water and ethanol several times, and then dried in an infrared oven at 90 °C. The precipitation reaction of the chemical reagents was determined by the following chemical equation:

(1 − *x*) Sr(NO_3_)_2_ + *x* Er (NO_3_)_3_ + (2 + *x*) KF → Sr_1−*x*_Er*_x_*F_2+*x*_↓+ (2 + *x*) KNO_3_. (1)

The derived fluoride nanopowders were poured directly into a cavity on a graphite mold with a diameter of 22 mm. The powder and graphite mold were separated by a graphite sheet coated with boron nitride. The specimen was heated at different temperatures ranging from 500 to 700 °C for each different soaking time with a pressure of 60 MPa under a vacuum degree of 10^−3^ Pa. After sintering, the ceramic samples were mirror-polished on both sides. The final thickness of the samples was 2.92 mm. 

### 2.2. Characterization

Phase structure of the powder and sintered ceramics was detected by X-ray diffraction (XRD, D2, Bruker, Karlsruhe, Germany) with a monochromatized source of Cu Kα radiation (λ_em_ = 0.1541 nm) in the range 20° ≤ 2θ ≤ 90°. Morphology of the nanoparticles was recorded by field emission scanning electron microscopy (FESEM, S4800, Hitachi, Tokyo, Japan). Microstructure of the fracture surface of the sintered ceramics was imaged by scanning electron microscopy (SEM, JSM-6510, JEOL, Tokyo, Japan) with an instrument equipped with a tungsten filament. The optical transmittance of the Er:SrF_2_ transparent ceramics was tested by an ultraviolet-visible-near-infrared (UV-VIS-NIR) spectrophotometer (Lambda 950, Perkin-Elmer, Waltham, MA, USA). Photoluminescence emission spectra of the 5 at. % Er^3+^ doped SrF_2_ transparent ceramics were recorded using a fluorescence spectrometer (FS980, Edinburgh Instruments, Edinburgh, UK) equipped with a monochromator and a steady InSb detector cooled with liquid nitrogen. A 975-nm laser diode (Wavespectrum Laser Group Ltd., Hefei, China) was used as the excitation source. Density of the sintered ceramics was measured by Archimedes’ Principle. The average grain size of the ceramics was obtained using the mean linear intercept method. The mean linear intercept method was carried out by the Nano Measurer software. All grain in the images were counted by the Nano Measurer software.

## 3. Results and Discussion

**Figure 1:**
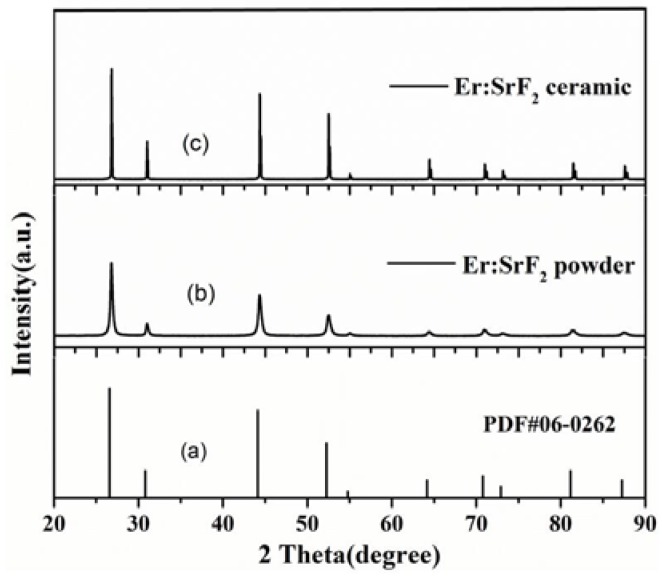
XRD patterns of Er:SrF_2_ powder and ceramics, sintered at 700 °C for 2 h. (**a**) The standard PDF pattern of SrF_2_; (**b**) Er:SrF_2_ powder; (**c**) Er:SrF_2_ ceramic.

Figure 1 shows the XRD patterns of chemically derived Er:SrF_2_ powder and ceramics when sintered at 700 °C for 2 h. We found that all the diffraction peaks were consistent with the cubic SrF_2_ standard patterns for both the powder and sintered ceramic. These results indicated that the powder and ceramic were pure, and that no second phase existed within samples. Furthermore, the Er^3+^ ions were also dissolved in the SrF_2_ crystal lattice. However, the difference was also presented in the XRD patterns. The widths of the diffraction peaks were different between powder and ceramics, which indicated that grain size and degree of crystallinity were diverse. After sintering, the grain sizes of the samples grew, in accordance with the narrowing of the diffraction peaks in the XRD patterns. Furthermore, the diffraction intensity was also enhanced through sintering, which was due to the increased degree of crystallinity of the samples.

**Figure 2:**
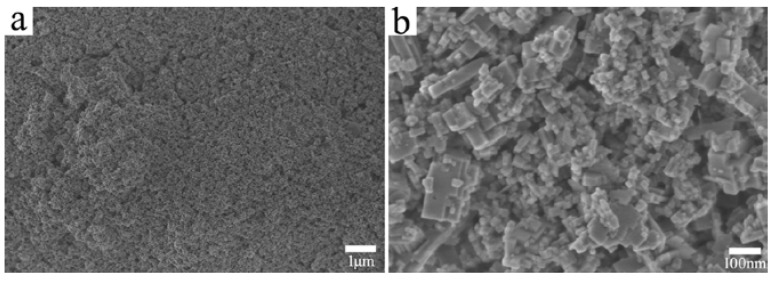
SEM images of the derived powder under different magnifications. (**a**) 10,000× magnification; (**b**) 100,000× magnification.

Figure 2 shows SEM images of the precipitated nanopowder at different magnifications. The SEM image in Figure 2a was obtained under 10,000× magnification, it shows that the particle-size distribution of the powder is homogenous. In contrast, the SEM image captured under 100,000× magnification revealed that the particle sizes are non-uniform. The sizes of the small particles were approximately 50 nm, while the sizes of the large particles were approximately 150 nm. The shapes of the particles were close to cubic. Fortunately, the sintering process was completed by hot-pressing. In pressureless sintering, the shape of the powder, especially the particle size of the powder, has a great influence on the sintering behavior. However, the influence of the properties of the powder on sintering behavior is weaker in hot-pressing sintering. 

**Figure 3:**
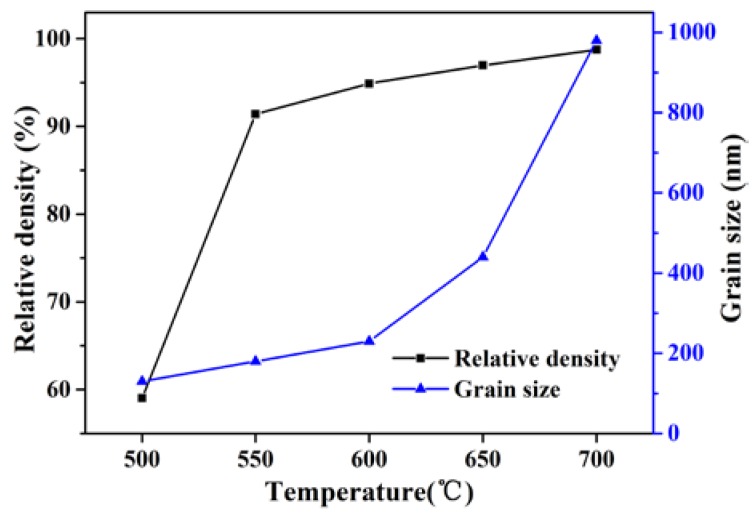
Densification and grain growth behavior of Er:SrF_2_ ceramic, sintered at 500–700 °C for 2 h.

**Figure 4:**
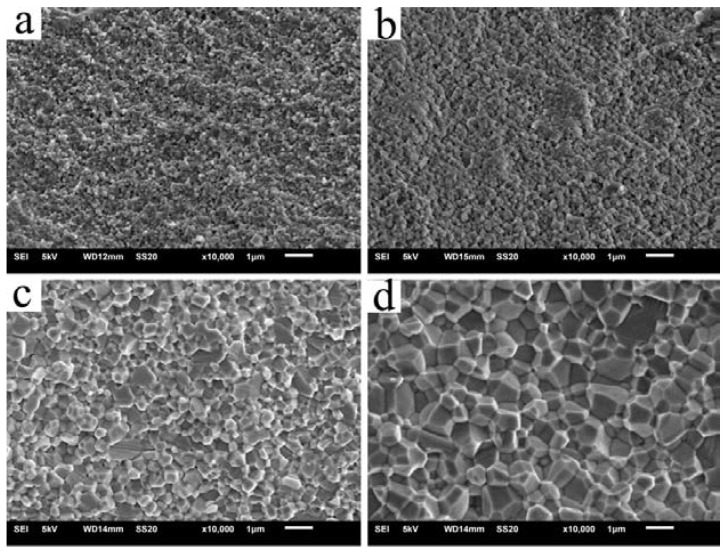
SEM images of Er:SrF_2_ sintered for 2 h at (**a**) 550 °C; (**b**) 600 °C; (**c**) 650 °C; and (**d**) 700 °C.

**Figure 5:**
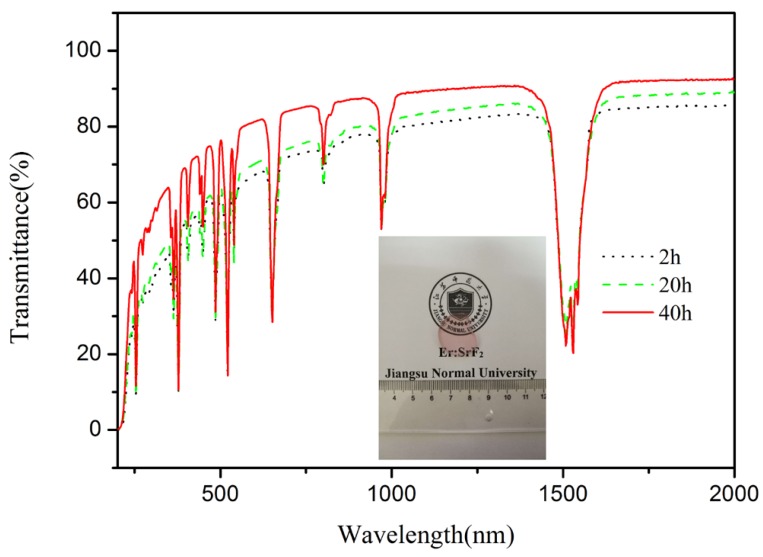
In-line transmittance of 5 at. % Er:SrF_2_ transparent ceramic, sintered at 700 °C for different holding times. Inset: photograph of 5 at. % Er:SrF_2_ transparent ceramic, sintered at 700 °C for 40 h.

Figure 3 presents the relative density and average grain size for the Er:SrF_2_ ceramic, sintered between 500 and 700 °C for 2 h. The relative density of the ceramic increased by increasing the sintering temperature. In particular, the relative density of the sintered body was only 59% at a sintering temperature of 500 °C. This relative density was very close to the green body of the samples after cold isostatic pressing (CIP), and the average grain size of the sintered body was approximately 150 nm. All the results show that the green body did not begin to densify at 500 °C, and that many open porosities existed in the sintered body. However, the relative density of the sintered body increased sharply to 92% when the sintering temperature reached 550 °C. As shown in Figure 4a at 550 °C, the porosity closed, and its average grain size was 180 nm. With increasing sintering temperature, the increase in relative density was slow. For the average grain size of the sintered body, there was virtually no grain growth before 600 °C. When the sintering temperature increased from 600 to 700 °C, the average grain size increased from 220 to 980 nm, as shown in Figure 4. Furthermore, the relative density of the sintered body was close to 99% when the sintering temperature reached 700 °C. Figure 4 shows that pores were removed as sintering temperature increased. No obvious pores were observed at 700 °C. The transmittance of the 5 at. % Er:SrF_2_ transparent ceramic, when sintered at 700 °C for 2 h, is plotted in Figure 5. The highest in-line transmittance reached 82%. However, the transmittance in the visible range decayed quickly, which can be attributed to the absorption of the Er^3+^ ions, and the nanoscale residual pores in the ceramic [39]. The nanoscale residual pores may exist in the triangulation boundary, which can only be observed with a high-magnification electron microscope.

To improve the optical quality of the transparent ceramic, the microstructure evolution of the ceramic, and the densification mechanism, must be clarified further. In particular, the atom diffusion mechanism in the final-stage sintering must be revealed. Therefore, the 5 at. % Er:SrF_2_ transparent ceramic was fabricated by hot-pressing at 700 °C for different times. 

During final stage sintering, the grain growth can be depicted by the grain growth kinetic equation model [14,40].

(2) $$G^{n}-G_{0}^{n}=kt,$$

where *G* is the average grain size at time *t*, *G*_0_ is taken to be the grain size at 2 h holding time, *k* is a rate constant at a certain temperature, and *t* is holding time. The grain growth data were fitted to *n* values of 2–4. As described in the literature, a grain growth exponent of 2 indicates grain boundary diffusion, *n* = 3 indicates lattice diffusion, and *n* = 4 indicates the surface diffusion [41].

**Figure 6:**
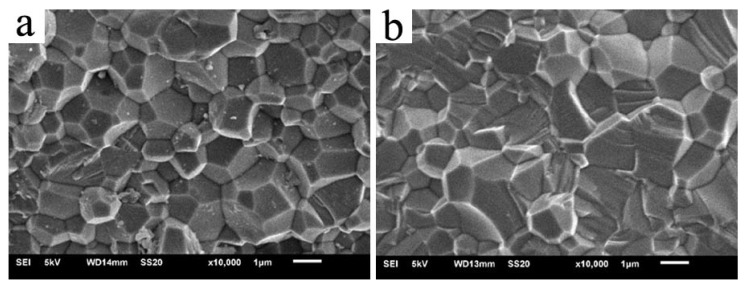
SEM images of Er:SrF_2_ sintered at 700 °C for 20 h (**a**) and 40 h (**b**).

**Figure 7:**
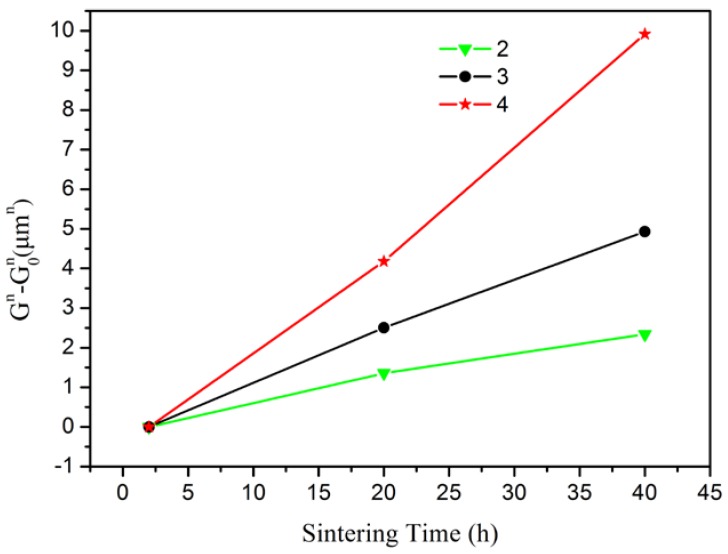
$G^{n}-G_{0}^{n}$ vs. sintering time at 700 °C, for Er:SrF_2_ ceramic at different holding times.

To clarify the grain growth mechanism, the grain sizes of the ceramics sintered at 700 °C were counted. Figure 6 shows SEM images of the 5 at. % Er:SrF_2_ sintered at 700 °C for 20 h and 40 h with average grain sizes of 1.49 and 1.79 μm, respectively. The grain size data were fitted by an exponent of 2, 3 and 4, respectively, as shown in Figure 7. The results show that the linear regressions with an exponent of 3 were the best. Thus, we know that lattice diffusion is the grain growth mechanism in the 5 at. % Er:SrF_2_ system.

Furthermore, the in-line transmittance of the transparent Er:SrF_2_ ceramics sintered for different times were operated ranged from 200 to 2000 nm. Figure 5 shows the in-line transmittance of the 5 at. % Er:SrF_2_ transparent ceramic, sintered at 700 °C for different holding times. The in-line transmittance increased with an increase in holding times. It is worth noting that the transmittance in the visible region was distinctly increased, as shown in Figure 5. The highest transmittance of the ceramic, sintered at 700 °C for 40 h, reached 92%, which is very close to the theoretical value of the SrF_2_ single crystal. This indicates that nanopores at the grain boundary were further excluded along with grain growth for long holding times. However, despite the obvious improvement of the in-line transmittance of the ceramic in ultraviolet and visible ranges, it can still be further optimized. Prolonging the soaking time is one way to improve the optical quality of the ceramic, but the time cost is not efficient. From Figure 2b, we can see that the dispersity of the nanoparticle powder may not be satisfactory. Agglomeration, especially hard agglomeration, may exist in the powders. The pores between the hard agglomeration powders, which were difficult to exclude, will form nanopores in the grain boundary. As a result, the synthesis of the monodisperse Er:SrF_2_ nanopowder, or, deagglomeration of the nanopowder, is the ultimate route to improving the in-line transmittance in the ultraviolet-visible region. Accordingly, in the inset of Figure 5, a photograph of the 5 at. % Er:SrF_2_ ceramic, sintered at 700 °C for 40 h, can be clearly seen. 

**Figure 8:**
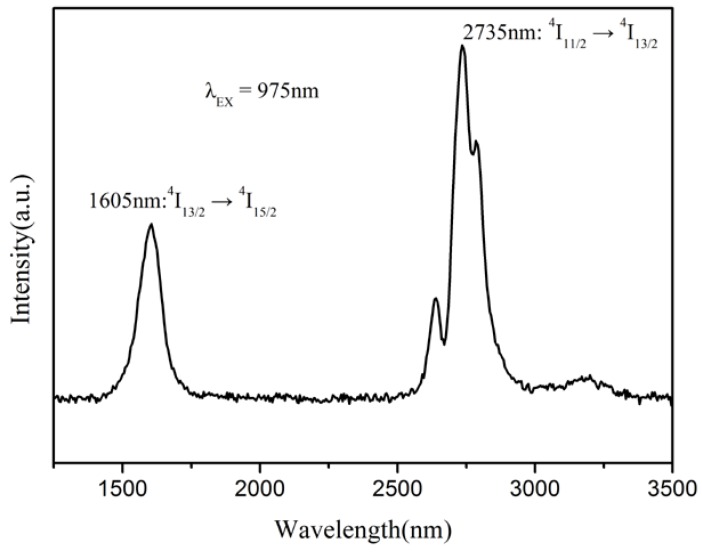
Infrared emission spectra of Er:SrF_2_ transparent ceramic, ranging from 1200 nm–3500 nm for an excitation of 975 nm, at room temperature.

As shown in Figure 8, the room temperature emission spectra ranged from 1200 to 3500 nm for a 5 at. % Er:SrF_2_ transparent ceramic, sintered at 700 °C for 40 h. It was recorded under an excitation wavelength of 975 nm. The strongest band was centered at 2735 nm, which can be attributed to ^4^I_11/2_ → ^4^I_13/2_ transition of Er^3+^ ions. These results verify that it is possible to achieve a laser output of approximately 2.7 μm in 5 at. % Er^3+^ ions doped SrF_2_ transparent ceramics. 

## 4. Conclusions

High quality 5 at. % Er:SrF_2_ transparent ceramics were fabricated by hot-pressing at 700 °C for 40 h using chemically-derived powder. Furthermore, the densification and microstructure evolution of the Er:SrF_2_ ceramics were systematically investigated. In addition, the grain growth kinetics of Er:SrF_2_ were clarified. This is the first time, to our knowledge, that lattice diffusion was confirmed as the grain growth mechanism of the Er:SrF_2_ transparent ceramics. The highest in-line transmittance of the Er:SrF_2_ transparent ceramics reached 92% at 2000 nm, which is very close to the theoretical transmittance value of the SrF_2_ single crystal. Furthermore, the Er:SrF_2_ transparent ceramic emission spectra showed the strongest emission band at 2735 nm, which means that it is possible to achieve a laser output of approximately 2.7 μm in 5 at. % Er^3+^ ions doped SrF_2_ transparent ceramics.
